# 444. County-level COVID-19 Case Fatality Rate in Medicaid Expansion States Compared to Non-Expansion States

**DOI:** 10.1093/ofid/ofab466.643

**Published:** 2021-12-04

**Authors:** Walid El-Nahal, Stephen Berry, Kevin Psoter, Kelly Gebo

**Affiliations:** 1 Johns Hopkins University School of Medicine, Baltimore, Maryland; 2 Johns Hopkins, Baltimore, MD

## Abstract

**Background:**

Medicaid expansion has been adopted by 38 states and the District of Columbia,^1,2^ contributing to lower rates of uninsured individuals in the US.^3^ During the COVID-19 pandemic, Medicaid enrollment offset employer-based insurance losses precipitated by the recession.^4^ The aim of this study was to evaluate whether Medicaid expansion may have impacted COVID-19 mortality.

**Methods:**

We conducted an ecologic study that included all US counties in the 50 states and District of Columbia. County-specific Medicaid expansion status was based on whether expansion was adopted within the state. COVID-19 cases and deaths for each county were obtained from the Centers of Disease Control (CDC). Unadjusted and multivariable negative binomial regression with robust standard errors to account for clustering of counties within each state were used to evaluate the association of COVID-19 case fatality rate and Medicaid expansion status. Adjusted models included the addition of four sets of county-level covariates thought to influence the association of Medicaid status and COVID-19 fatality rate: demographics, comorbidities, economic indicators, and physician density. These analyses were then performed in subgroups of counties defined by urbanicity (metro, suburban or rural) and quartiles of poverty rates. Incidence Rate Ratios (IRR) and 95% confidence intervals (CI) are reported.

**Results:**

A total of 1,814 Medicaid expansion and 1,328 non-expansion counties were included in the analysis. Crude case fatality rates were 2.1% (non-expansion) and 1.8% (expansion). Medicaid expansion was not associated with a significantly lower COVID-19 case fatality rate in either the unadjusted (IRR: 0.86; 95% CI: 0.74, 1.01) or fully adjusted (IRR: 1.02; 95% CI: 0.90, 1.16) models. In adjusted models, Medicaid expansion status was also not associated with differences in COVID-19 case fatality rate when counties were stratified by either urbanicity or percent of individuals living below the poverty line.

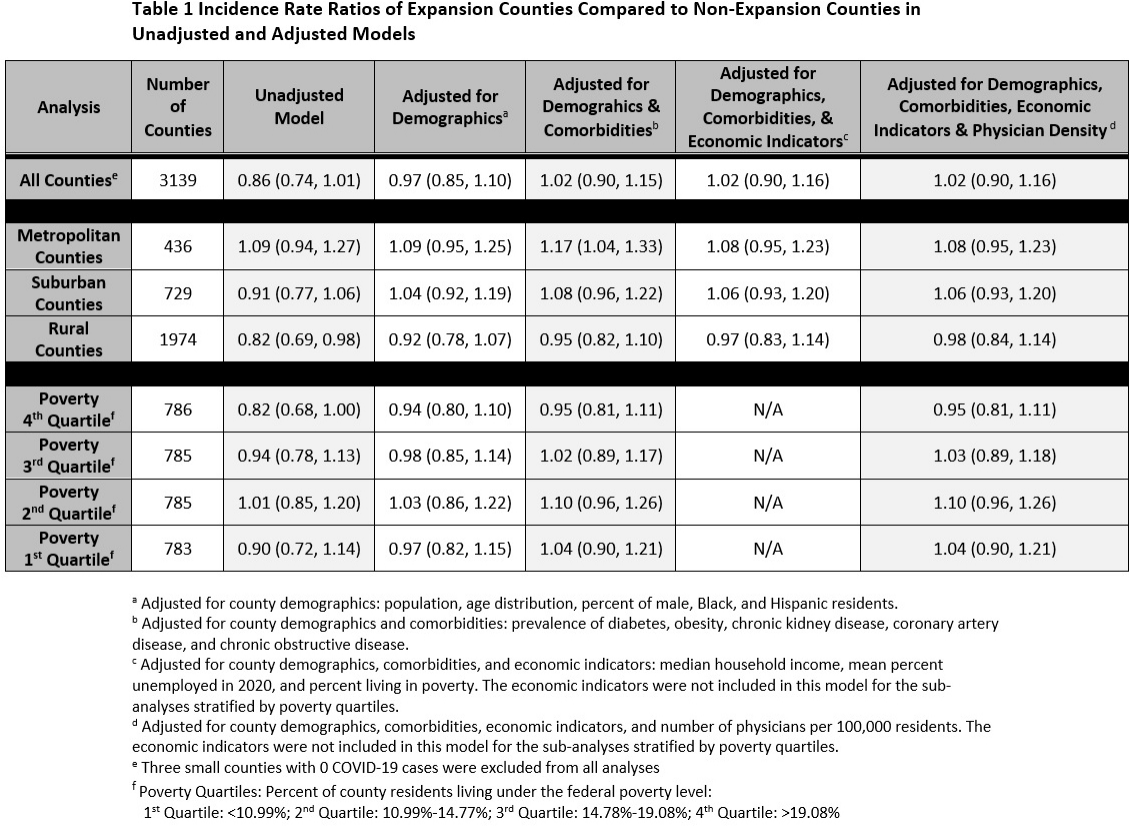

**Conclusion:**

In this county-level analysis, Medicaid expansion status was not associated with a significant difference in county-level COVID-19-related case fatality rates among people of all ages. Future individual-level studies are needed to better characterize the effect of Medicaid on COVID-19 mortality.

**Disclosures:**

**All Authors**: No reported disclosures

